# Expression of Placenta growth factor (PlGF) in non-Small cell Lung cancer (NSCLC) and the clinical and prognostic significance

**DOI:** 10.1186/1477-7819-3-68

**Published:** 2005-10-13

**Authors:** Lijian Zhang, Jinfeng Chen, Yang Ke, Robert E Mansel, Wen G Jiang

**Affiliations:** 1 Department of Surgery, Peking University School of Oncology, No. 52 Fu-cheng Road, Haidian District, Beijing, China; 2 Department of Cell Biology, Health Science Center, Peking University, No. 38 Xueyuan Rd, Hai Dian District, Beijing China; 3 Metastasis & Angiogenesis Research Group, Wales College of Medicine, Cardiff University, Cardiff, UK

## Abstract

**Background:**

Placenta growth factor (PlGF) is a member of the vascular endothelial growth factor (VEGF) family. Over-expression of PlGF is known to be associated with pathological angiogenesis. This study examined PlGF expression at protein and message levels in non-small cell lung cancer (NSCLC), in which no reports on the significance of PlGF expression is available to date.

**Patients and methods:**

We used immunohistochemistry to assess the PlGF protein and correlated PlGF with microvessel density (MVD), as well as clinical outcome in patients with NSCLC tumours (n = 91). In addition, we applied a real time quantitative PCR assay using SYBR Green chemistry to measure PlGF mRNA in normal lung tissues and NSCLC tumours.

**Results:**

PlGF was positively stained mainly in cytoplasm of lung cancer cells. High level staining of PlGF was found in 38.5% NSCLC patients. A high level of MVD in NSCLC was found in 42.9% of cases. Tumours with high level and low level PlGF staining had a significantly different MVD (26.69 vs. 20.79, respectively, p = 0.003). Using both univariate and multivariate analyses, PlGF was found to be an independent prognostic factor. Real time PCR analysis revealed that PlGF mRNA was higher in the cancer tissue than normal tissue (0.95 ± 0.19 vs. 0.57 ± 0.24; p < 0.005) and that PlGF mRNA was significant higher in III-IV stage patients than in I-II stage patients (1.03 ± 0.20 vs. 0.80 ± 0.17; p = 0.011).

**Conclusion:**

PlGF expression is significantly more in NSCLC tumour tissues than in matched normal tissues. It has a significant positive association with MVD and is an independent factor for NSCLC patients. PlGF may have a pivotal role in NSCLC development and disease progression.

## Introduction

Angiogenesis is essential for a solid tumour to grow beyond 1–3 mm in diameter [1]. It also is a significant predictive factor for prognosis in patients with solid tumours [2,3]. Amongst the numerous angiogenic factors, VEGF is the most powerful and most extensively studied. VEGF belongs to a protein family, within which Placental growth factor (PlGF) is a member (other members include VEGF-B, -C and D). PlGF is a secreted, disulfide-linked dimeric glycoprotein originally cloned from a cDNA library of term placenta [4]. PlGF shares 53% of similarity in its overall amino acid (aa) residues with VEGF. The biological functions of VEGF and PlGF are similar, including stimulation of the growth of vascular endothelial cells [5]. As a result of alternative splicing of the primary PlGF transcript, PlGF has at least three isoforms, PlGF-1 (PlGF149), PlGF-2 (PlGF170) and PlGF-3 (PlGF221) [4]. In cells co-expressing VEGF and PlGF mRNA, a heterodimeric VEGF/PlGF protein has been detected [6,7]. VEGF/PlGF heterodimer has been shown to promote capillary growth *in vivo *[7].

PlGF is known to specifically bind with Flt-1. VEGFR2/KDR/Flk-1 and VEGFR1/Flt-1 are the two main receptors of VEGF during the embryonic vascular development [8,9]. Flk-1 primarily mediates VEGF signal transduction and biological responses [10]. In addition to acting as the receptor for VEGF and PlGF, Flt-1 is a special receptor for VEGF-B. It has been shown that VEGF and PlGF can induce transcription factors FosB and c-Fos mRNA expression, indicating the possibility that these factors may play a role in the biological responses mediated by PlGF and Flt-1 [9]. The protein and message for PlGF can be detected in endothelial and epithelial cells and have been found in a few tumours [7,10].

To our knowledge, there has been no report on the significance of PlGF expression with clinical outcome of patients with lung cancer, including non-small cell lung cancer (NSCLC). In order to ascertain the clinical significance of PlGF expression in human non-small cell lung cancer, we analysed the expression pattern of PlGF using both immunohistochemical method and real time quantitative PCR and attempted to establish if a relationship existed between PlGF and MVD, and subsequently between PlGF and the predicted prognosis.

## Patients and methods

### Patients and samples

A total of 91 patients with non-small cell lung cancer, who attended Beijing Cancer Hospital from July 2000 to August 2003, were included. None of the patients received any neoadjuvant therapy prior to operation. Histological types of the lung cancer included squamous carcinoma, adenocarcinoma, large cell carcinoma, squamous adenocarcinoma and alveolar carcinoma, pathologically (table 1). No other previous or concomitant primary cancer was present. Clinico-pathological characteristics were defined according to the TNM criteria of the UICC (11) (table 1). Slides were reviewed and evaluated by two independent researchers. Clinico-pathologic factors, such as age, sex, histological types of tumours, tumour cell grade, TNM stage, vessel embolism, lymph node metastasis, were reviewed and stored in the patients' database. Patients were followed up from the day of operation to August 2004 as the end of follow-up. The follow-up intervals were calculated as survival intervals after surgery.

**Table 1:** The correlation between PlGF expression and the clinical pathological factors.

**Variable**	**Case(n and %)**	**PlGF staining pattern**		**P value (*x*^2^)**
			
		**Low-staining (n =)**	**High-staining (n =)**	
**Sex**				
Male	63 (69.2%)	39	24	1.000
Female	28 (30.8%)	17	11	

**Age**				
≤60	43 (47.3%)	29	14	0.290
>60	48 (52.7%)	27	21	

**Histological type**				
Squamous carcinoma	42 (46.2%)	29	13	0.167
Adenocarcinoma	33 (36.3%)	20	13	
Adenosquamous ca.	6 (6.6%)	3	3	
Large cell carcinoma	2 (2.2%)	2	0	
Carcinoid	2 (2.2%)	0	2	
Alveolar carcinoma	6 (6.6%)	2	4	

**Grade of differentiation**				
Poor	11 (12.1%)	9	2	0.288
Moderate	48 (51.7%)	27	21	
Well	32 (35.2%)	20	12	

**Tumour stage**				
T1	9 (9.9%)	7	2	0.643
T2	60 (65.9%)	37	23	
T3	19 (20.9%)	10	9	
T4	3 (3.3%)	2	1	

**Nodal status**				
N (-)	49 (53.8%)	31	18	0.829
N (+)	42 (46.2%)	25	17	

**Vessel cancer embolus**				
V (-)	67 (73.6%)	42	25	0.808
V (+)	24 (28.4%)	14	10	

**TNM stage**				
I	40 (43.9%)	28	12	0.173
II	20 (21.9%)	11	9	
IIIa	28 (30.7%)	17	11	
IIIb	1 (1.1%)	0	1	
IV	2 (2.2%)	0	2	

A separate collection of tissue samples from 21 primary non-small cell lung cancers were used for mRNA based analysis. These tumours were resected surgically from patients at the Clinical Oncology School of Peking University from 2002 to 2003 and were saved in the Tissue Bank of Peking University Oncology School. The patients consisted of 13 men and 8 women, with a mean age of 56.2 ± 6.4 years. The histological type of lung cancer was classified based according to the World Health Organization classification [12]. Tumour staging was performed according to the TNM staging criteria of the UICC [11]. The tumour specimens included 10 squamous cell carcinomas, 8 adenocarcinomas, and 3 undifferentiated carcinomas. Tumour staging was I in 3 cases, II in 7 cases, IIIA or IIIB in 10 cases, and IV in 1 case. Immediately after surgery, tumour samples and surrounding normal lung tissues (more than 5 cm away from the tumour margin) were placed in liquid nitrogen and stored frozen at -80°C for RNA extraction and the RT-PCR.

### Materials

The goat polyclonal antibody of PlGF and a mouse monoclonal antibody of β-actin was purchased from Santa Cruz Biotechnology (Santa Cruz, California, USA). The mouse monoclonal antibody of CD31 was purchased from Beijing Zhongshan Biotechnology Co. Ltd (Beijing, China). The biotin conjugated anti-goat IgG, anti-mouse IgG antibodies were purchased from Sigma (Poole, Dorset, England, UK). The Horse Radish Peroxidase (HRP) conjugated anti-goat IgG, anti-mouse IgG antibodies were obtained from Sigma (Poole, Dorset, England, UK). The Target Retrieval Solution was purchased from DAKO Corp. (Beijing, China). RNA extraction and reverse transcription kits and PCR mix were purchased from Bio-Rad Corp. (Beijing, China). Primers were synthesized by BioAsia Corporation (Shanghai, China). PCR reaction was carried out using the iCycler iQ™ system (Bio-Rad). The working stock solution of SYBR Green is 1:100 (Bio-Rad).

### PlGF staining and microvessel counting

The paraffin-embedded tissue sections of 91 patients were cut at 4 microns and mounted on polylysin-coated glass slides for immunohistochemistry. Briefly, deparaffinized sections were heated (60°C 1 hour). Antigen retrieval was performed by heating the samples without boiling in Target Retrieval Solution (DAKO Corp.), pH 6.70 (200 ml) in a microwave oven for 10 min. After endogenous peroxidase was blocked with 3% hydrogen peroxide in the section, each section was incubated with non-immunised horse serum (Sigma) for 15 minutes, in order to block the non-specific antigen site.

The immunohistochemical staining procedure was performed according to the protocol of the DAKO Corp. The primary anti-PlGF antibodies were used at a dilution 1:100 from the stock. The primary CD31 antibody was used at working dilution of 1:100. The specificity of anti-PlGF antibody was documented elsewhere [13]. Following incubation at 4°C overnight, the sections were extensively washed and then incubated with link antibodies (Sigma). Following more washing, bound antibodies were linked to avidin-biotin-peroxidase according to manufacturer's instruction (Dako Corp.). The staining was completed by developing colour using the DAB (diaminobenzidine tetrahydrochloride) solution for 5 min. The slides were counterstained with Mayers Haematoxylin Blue in 0.3% ammonia. For negative controls, sections were stained in the same manner, except that the primary antibody was absent from the solution.

PlGF staining in lung cancer cells was independently assessed by two observers using a modification of the system of grading the relative intensity of immunoreactivity for the respective antibodies [14]. PlGF immunohistochemical staining of a tissue sample was graded as either low level expression or high level. High-level staining represented uniformly intense immunoreactivity; low level staining represented patchy and weak or negative immunoreactivity.

MVD was evaluated as previously described [15]. After screening the areas with intense neovasularized spots at low power field (×100), microvessels in the area with the highest number of discrete microvessels were counted in a ×400 field. Three separate intense neovascularized areas were assessed for each spot, and the mean was calculated as MVD of each tumour evaluated. The MVD level were graded as low level with MVD number lesser than 26, while the high level with MVD number over than 26. This was based on the pilot analysis of the microvessel density according to pathological grade, in that 26 yielded a clear division between the groups.

### Generation of cDNA from NSCLC tissue and normal tissue and RT-PCR

RNA was extracted from tumour and the normal surrounding tissues in RNA extraction buffer according to the manufacturer's protocol. The concentration of RNA was measured with a spectrophotometer. Reverse transcription was performed from 1 μg of total RNA using oligo dt primer according to the manufacture's instructions. Conventional PCR primers were designed using Primer 3 , to allow amplification of regions that have no overlap with other known genes and span at least one intron. Primers were synthesized by BioAsia Corporation (Shanghai, China): Primer sequences for PlGF were 5'ACGTGGAGCTGACGTTCTCT'3 and 5'CAGCAGGAGTCACTGAAGAG'3 and for GAPDH 5'AGGTCGGAGTCAACGGATTTG'3 and 5'GTGATGGCATGGACTGTGGT'3. Conventional PCR was performed using cDNA from tissues together with the PCR master mix using respective primers. The reaction conditions were: 95°C 5 min, 94°C for 1 min, 55°C for 45 s, 72°C for 30 s and a final extension phase at 72°C for 7 min for 40 cycles. The PCR products were separated on a 2% agarose gel and stained with 5 μl ethidium bromide prior to examination under UV light and photographs taken.

### Generation of standard for real time PCR analysis

The PCR product from the above reaction was gel-excised and purified using gel purification kit (Tianwei Corp, Beijing). It was subsequently quantified on a gel with lambda molecular weight standards and in a spectrophotometer. The number of copies of target template was calculated. The DNA sample was serially diluted to yield a concentration range between 10^2 ^to 10^8 ^copies, which was subsequently used as the internal standard. This was finally prepared in elution buffer, aliquoted and stored at -80°C until use.

### Real Time quantitative RT-PCR

The iCycler iQ™ system incorporates a gradient thermocycler and a 96-channel optical unit. SYBR Green as a DNA-dye can affinity dsDNA was used to detect the PCR product of PlGF and GAPDH. The melting point, optimal conditions and the specificity of the reaction were first determined using a standard procedure [17]. The working stock solution of SYBR Green is 1:100 (Bio-Rad). Quantitative PCR was carried out in 96-well plate with 10 pmol forward and reverse primers, and the working solution SYBR green, using a customer PCR master mix, with the following conditions: 95°C for 5 minutes, followed by 40 cycles at 95°C for 1 minute, 55°C for 45 seconds, 72°C for 30 second. Every assay included test cDNA samples, 10-fold serial dilutions of the standard qualification, and controls (no template), as we previously reported [16]. The copy number of each transcript was calculated as the relative copy number normalised by GAPDH copy number.

### Statistical analysis

Patients were divided into two groups: those with high-level PlGF staining and those with low-level staining. Chi-square analysis was used to test the association of PlGF expression level with standard pathological variables. Clinical pathological parameters and PlGF expression status were correlated with survival time in both univariate and multivariate analyses. Variables included in univariate analysis were gender, TNM stage, grade of differentiation, vessel cancer embolus, lymph nodal status and use of postoperative adjuvant therapy, MVD and PlGF. Variables included in multivariate analysis were MVD, PlGF expression status, gender, TNM stage, grade of differentiation, vessel cancer embolus, lymphatic nodal status and use of postoperative adjuvant therapy. The log-rank test was used to test equality across categorical factors in univariate analysis, and the level of significance was set at p ≤ 0.05 based on two sided test. The multivariate analysis was performed using Cox proportional hazards model, and the level of significance was set at p ≤ 0.05 based on a two sided test.

Paired-samples analysis was used (Student's *t*-test) to determine whether the difference of PlGF mRNA expression level observed between matched cancer tissue and the normal tissue. Independent-samples analysis was used (Student's *t*-test) to determine whether the differences observed between PlGF expression level in NSCLC tissue and the clinico-pathological characteristics. Statistical tests were performed using the software SPSS 10.0 (SPSS Inc., Chicago).

## Results

### Immunohistochemical analysis and the localization of PlGF in lung cancer

**Figure 1:**
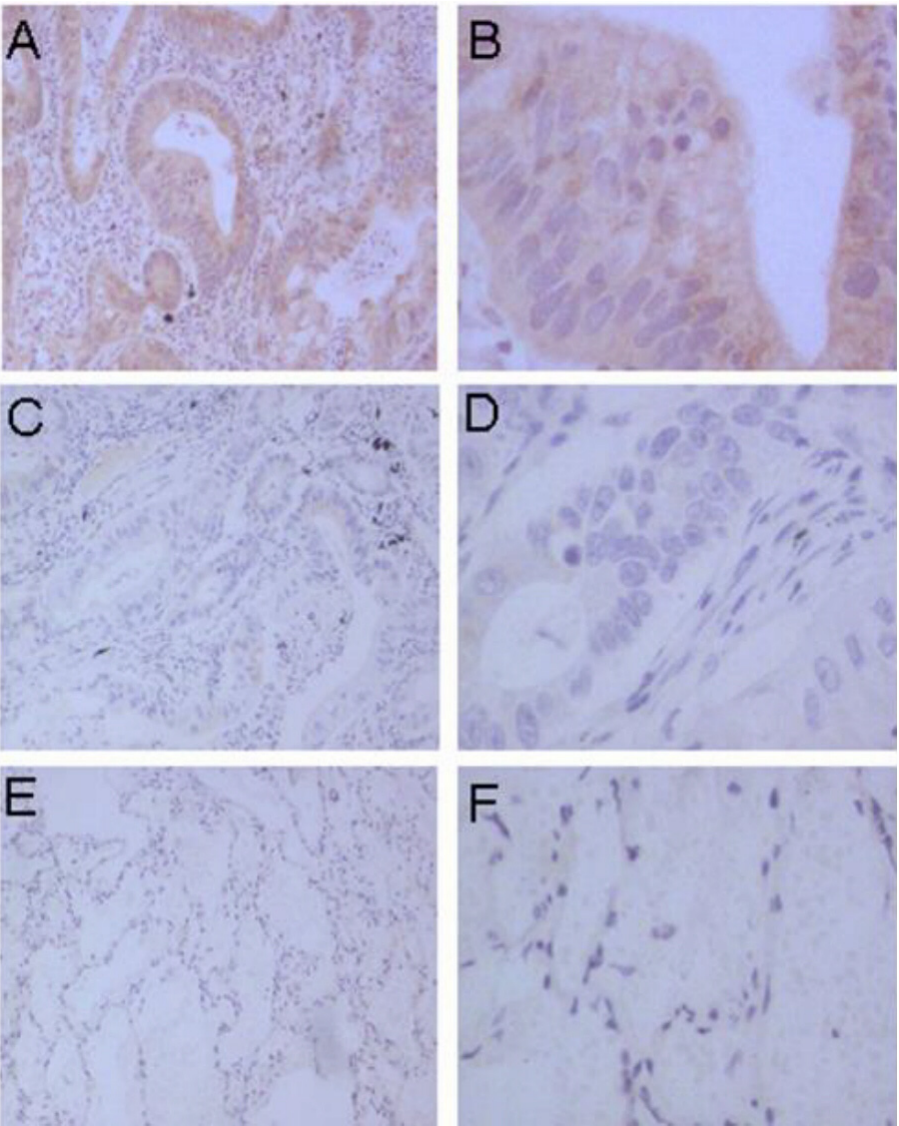
Immunohistochemical staining for PlGF in adenocarcinoma of lung. A and B showed strong diffuse cytoplasmic staining of PlGF in adenocarcinoma of lung. C and D showed the negative staining of PlGF in adenocarinoma of lung. E and F showed the negative staining status of PlGF in normal alveolar (Original magnification is ×100).

**Figure 2:**
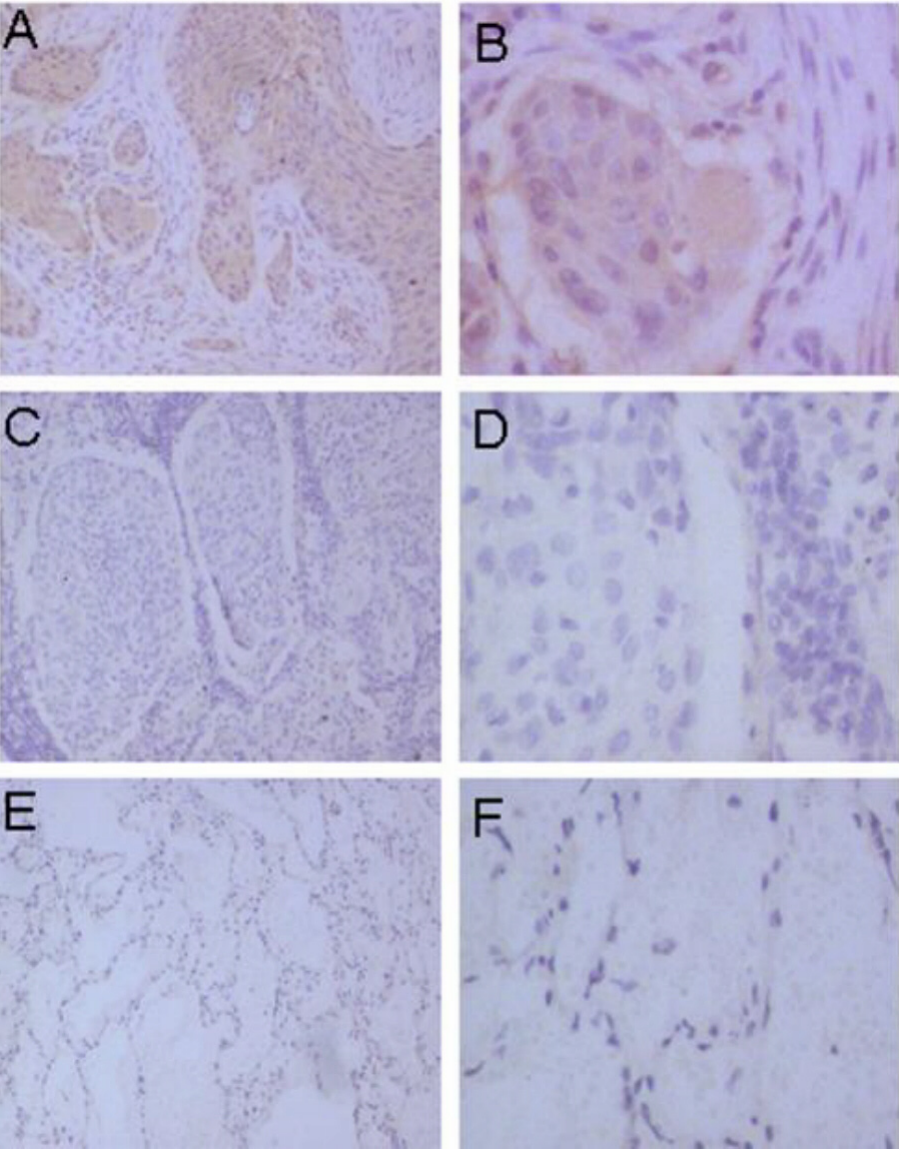
Immunohistochemical staining for PlGF in squamous cell carcinoma of the lung. A and B showed strong diffuse cytoplasmic staining of PlGF in squamous carcinoma of the lung. C and D showed negative staining of PlGF in squamous carcinoma of lung. E and F showed the negative staining status of PlGF in alveolar. (Original magnification ×400)

Lung cancer cells stained positively for PlGF. As shown in figure 1A, 1B, adenocarcinoma cells showed strong and diffuse cytoplasmic staining of PlGF. Squamous cancer cells of the lung also displayed strong and positive cytoplasmic staining of PlGF as shown in figure 2A, 2B. Compared with normal tissues of the lung as shown in figure 1E, 1F and 2E, 2F, the negative staining in tumour tissues was shown in figure 1C, 1D and 2C, 2D.

### PlGF and the clinical correlation

The immunohistochemical staining results of PlGF in NSCLC were shown in table 1. High level expression of PlGF in NSCLC was found in 35 (38.5%) cases. Among all the available information, no significant correlation was seen (p > 0.05).

### Micro-vessel Density in lung cancer and its correlation with PlGF

The micro-vessel endothelial cells stained positively for CD31. Endothelial cells showed strong and diffuse cytoplasmic staining of CD31, The correlation between MVD and clinico-pathologic features in NSCLC were shown in table 2. Low level of MVD in NSCLC was found in 52 (57.1%) cases. Amongst the available clinical parameters, no clinical factors showed significant correlation with CD31 as shown in table 2 (p > 0.05).

**Table 2:** Microvessel Density counting *vs*. clinico-pathological features in the complete series (n = 91)

**Variable**	**Cases (n and %)**	**MVD pattern**		**P value (*x*^2^)**
			
		**Low MVD (n =)**	**High MVD (n =)**	
**Sex**				
Male	63 (69.2%)	37	26	0.654
Female	28 (30.8%)	15	13	

**Age**				
≤60	43 (47.3%)	24	19	0.835
>60	48 (52.7%)	28	20	

**Histological type**				
Squamous carcinoma	42 (46.2%)	24	18	0.312
Adenocarcinoma	33 (36.3%)	20	13	
Adenosquamous carcinoma	6 (6.6%)	2	4	
Large cell carcinoma	2 (2.2%)	2	0	
Carcinoid	2 (2.2%)	0	2	
Alveolar carcinoma	6 (6.6%)	4	2	

**Grade of differentiation**				
Poor	11 (12.1%)	7	4	0.345
Moderate	48 (52.7%)	24	24	
Well	32 (35.2%)	21	11	

**Tumour stage**				
T1	9 (9.9%)	5	4	0.988
T2	60 (65.9%)	34	26	
T3	19 (20.9%)	11	8	
T4	3 (3.3%)	2	1	

**Nodal status**				
N (-)	49 (53.8%)	29	20	0.678
N (+)	42 (46.2%)	23	19	

**Vessel cancer embolus**				
V (-)	67 (73.6%)	39	28	0.812
V (+)	24 (26.4%)	13	11	

**TNM stage**				
I	40 (44.0%)	27	13	0.389
II	20 (22.0%)	10	10	
IIIa	28 (30.8%)	14	14	
IIIb	1 (1.1%)	0	1	
IV	2 (2.2%)	1	1	

35 lung NSCLC tumours that had high-level expression of PlGF had a mean MVD at 26.69 and the standard deviation (SD) was 8.89. While in 56 NSCLS tumour which had low-level staining of PlGF, the mean MVD ± SD was 20.79 ± 8.82. The difference between the two groups was highly significant, p = 0.003.

### Clinical outcome and the prognostic value of variables

Univariate analysis of the impact of histological types and PlGF expression status on prognosis was presented in table 3. Longer survival time was found to be significantly correlated with the following factors: low TNM stag (p = 0.0005), no lymph node metastasis (mean survival time, 41.04 months vs. 31.25 months, p = 0.0051), low level of MVD (mean survival time, 39.96 months vs 31.85 months, p = 0.0434), and PlGF low-level expression (mean survival time, 40.68 months vs 27.76 months, p = 0.0028).

**Table 3:** Potential prognostic factors using univariate analysis

**Characteristics**	**Patients (n =)**	**Mean survival (mths)**	P Value*
**Gender**			
Male	63	36.42 (31.77–41.08)	0.8860
Female	28	37.08 (30.73–43.44)	

**TNM stage**			
I	40	41.61 (37.35–45.87)	0.0005
II	20	38.44 (31.18–45.70)	
IIIa	28	30.31 (22.76–37.86)	
IIIb	1	6.00 (6.00-6.00)	
IV	2	13.50 (3.11–23.89)	

**Grade of differentiation**			
Well	32	37.43 (31.17–43.69)	0.4984
Moderate	48	34.76 (29.86–39.67)	
Poor	11	39.73 (28.34–51.12)	

**Nodal status**			
N (-)	49	41.04 (36.64–45.45)	0.0051
N (+)	42	31.25 (25.64–36.86)	

**Vessel cancer embolus**			
V (-)	67	37.50 (33.41–41.58)	
V (+)	24	33.89 (25.75–42.03)	

**Postoperative adjuvant therapy**			
No	26	37.47 (29.70–45.24)	0.7225
Chemotherapy or Radiation	65	36.03 (31.57–40.49)	

**MVD**			
High MVD	39	31.85 (25.68–38.03)	0.0434
Low MVD	52	39.96 (35.48–44.44)	

**PlGF staining**			
Low expression	56	40.68 (36.70–44.66)	0.0028
Over expression	35	27.76 (21.17–34.34)	

* Log-Rank Test

A multivariate prognostic analysis based on the Cox proportional hazard model was performed to test the independent value of each parameter predicting overall survival. Of the all the factors analysed (gender, tumour TNM stage, grade of differentiation, vascular emboli of cancer cells, nodal status and use of postoperative adjuvant therapy, MVD level, and PlGF), TNM stage and high-level PlGF were two independent prognostic factors, which were the best general prognostic indicators (p = 0.007 and p = 0.011, respectively) (table 4). Furthermore the relative risk of TNM stage and high-level PlGF expression were 1.311 and 2.738 respectively (95%CI, 1.076–1.597 *vs *1.269–6.102).

**Table 4:** The prognostic value of PlGF staining evaluated on the basis of multivariate analysis in the COX model (Backward: Wald) for 91 NSCLC cases.

**Variable**	**Regression Coeff. (B)**	**Standard error (SE)**	**Wald value**	**P value**	**OR (EXP)**	**95% CI for OR**
**PlGF expression**	1.024	0.401	6.532	0.011	2.738	(1.269–6.102)
**TNM staging**	0.271	0.101	7.201	0.007	1.311	(1.076–1.597)

**Figure 3:**
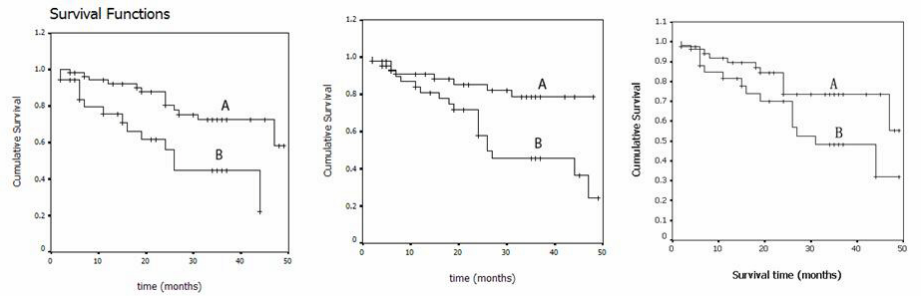
Overall survival of NSCLC patients based on PlGF (left), nodal status (middle) and MVD (right). Left: PlGF and the overall survival. A, PlGF low expression tumours (n = 56). B, PlGF over expression tumours (n = 35). The survival curves are significantly separated and the patients with PlGF low expression have long survival time than those with over expression (p = 0.0028, log-rank test). Middle: Overall survival based on nodal status of NSCLC patients (n = 91). The difference between nodal negative patients (A, n = 49) and nodal positive patients (B, n = 42) is significant (p = 0.0051, log-rank test). Right: Overall survival based on MVD status of NSCLC patients (n = 91). The difference between low level of MVD (A, n = 52) and high level MVD patients (B, n = 43) is significant (p = 0.0434, log-rank test).

Figure 3A shows the Kaplan-Meier survival curve, based on PlGF expression status. The cumulative survival time for patients with PlGF low-expression (n = 56) was significantly longer than the patients with high-level PlGF (n = 35) (p = 0.0028). Figure 3B shows the Kaplan-Meier survival curve for 91 NSCLC patients based on lymphatic node metastasis. The survival difference between lymph node negative patients (n = 49) and positive patients (n = 42) was significant (P = 0.0051). Figure 3C shows the Kaplan-Meier survival curve for 91 NSCLC patients based on MVD status. The survival difference between low level of MVD (n = 52) and high level MVD patients (n = 43) was significant (p = 0.0434).

### PlGF mRNA expression level in NSCLC and normal lung tissue assessed by real time RT-PCR

The amplification plot and the melting curve confirmed that the amplification products for all three molecules were specific (data not shown). We then employed the analysis tool for both PlGF and GAPDH. The PlGF mRNA (copy number of PlGF mRNA/copy number of GAPDH mRNA) is shown here as the relative copy number. PlGF mRNA expression and was detected in all 21 (100%) paired lung cancer and non-tumorous lung tissue samples by real time RT-PCR. The relative copy number for PlGF in 21 samples of lung cancer tissue ranged from 0.52 to 1.19, with a mean ± standard deviation (SD) of 0.95 ± 0.20, while the corresponding values in the matched non tumours lung tissue ranged from 0.21 to 1.00, with a mean ± SD of 0.57 ± 0.24. In 17 of 21 (81%) of patients, tumours displayed higher PlGF than the matched normal tissues. Only in 19% (4/21) of patients, healthy tissues had higher levels than tumour tissues. PlGF mRNA expression in lung cancer tissue was significantly higher than in the matched non tumorous lung tissue (95% CI: 0.2382~0.5321, p < 0.005, paired test).

### Relationship between PlGF mRNA expression and clinicopathologic variables

Table 5 shows the relationship between PlGF mRNA expression level and the clinico-pathological features. PlGF mRNA was significantly higher in III-IV stage patients than in stage I-II patients (1.03 ± 0.20 vs. 0.80 ± 0.17; p = 0.011, independent *t *test). The difference between PlGF in different tumour size was not significant (p > 0.05). The relationship between PlGF mRNA expression and sex, histological type, lymph node status was otherwise not statistically significant.

**Table 5:** Relationship between relative levels of PlGF mRNA (PlGF/G3PDH) and clinico-pathologic characteristics

**Variables**	**No.**	**PlGF mRNA^a ^(mean ± SD)**	***P *value**
**Sex**			
Male	13	0.8760 ± 0.2468	0.261
Female	8	0.9881 ± 0.1461	
**Histologic type**			
Squamous	10	0.9706 ± 0.2153	0.307
Non squamous	11	0.8714 ± 0.2177	
**Stage**			
I–II	10	0.7979 ± 0.1744	**0.011**
III–IV	11	1.0284 ± 0.1977	
**Tumour status**			
T1	6	0.7939 ± 0.1656	0.096
T2–4	15	0.9686 ± 0.2195	
**Lymph node status**			
N0	8	0.8231 ± 0.1640	0.116
N1–3	13	0.9775 ± 0.2303	
**Tissue**			
Tumour	21	0.9187 ± 0.2171	<0.005*
Adjacent normal tissue	21	0.5652 ± 0.2406	

^a^PlGF mRNA expression derived from real-time quantitative RT-PCR: PlGF/GAPDH.

*p value derived from paired *t *test; others derived from independent *t *test.

## Discussion

PlGF is a member of the VEGF family and is known to be a powerful angiogenic factor, like its family members VEGF. Although PlGF has been studies in a number of clinical tumour types, little is known with regard to this factor in lung cancer, particularly non-small cell lung cancer (NSCLC). The current study investigated the expression of PlGF, at the protein level and the messenger RNA level and whether it had a bearing on clinical outcome in patients with NSCLC.

Firstly, the current study has demonstrated that PlGF can be found, at the protein level and mRNA level, in lung cancer cells. Furthermore, PlGF protein immunostaining is primarily seen in the cytoplasmic region of the cells, which is in accordance with literature reports. Stromal cells and endothelial cells displayed little staining. Secondly, PlGF is significantly linked to MVD. High levels of PlGF are significantly linked to high MVD.

The angiogenic role of PlGF is interesting to observe. PlGF expression is restricted to the placenta tissue in normal physiological condition [18] and has been indicated to play an important role in the angiogenic process. There is evidence to suggest that up-regulation of PlGF and VEGFR-1 expression can stimulate the response of vascular endothelial cells to VEGF and enhance angiogenesis in pathological disorders [19]. Although the precise mechanism by which PlGF regulates angiogenesis is unclear, some leads have been suggested. Up-regulation of PlGF can displace VEGF from Flt-1 and will make more VEGF to bind and activate Flk-1 [20]. PlGF activates Flt-1 and will lead to intermolecular transphosphorylation of Flk-1. This can enhance phosphorylation levels of Flk-1 on tyrosine residues. Furthermore, VEGF/PlGF heterodimer can activate and transmit angiogenic signals through the Flk-1/Flt-1 heterodimer receptor complex.

In the current study, a high level of PlGF was seen 38.46% of all the cases. The significant correlation between PlGF and MVD further indicates that PlGF expression level is associated with MVD, and potentially with angiogenesis in human lung cancer. We, and others, have previously reported that MVD was an influence factor to predict the prognosis of patients with non-small cell lung cancer Furthermore, both univariate and multivariate analyses revealed that PlGF is an independent prognostic factor. Taken together, it is concluded that PlGF is an important factor that can influence the angiogenesis process in NSCLC, and that the levels of PlGF reflect MVD, which might be a predictive factor for NSCLC patients' prognosis.

The current study has used quantitative real time PCR, to determine the levels of PlGF gene transcript. It has been shown that PlGF mRNA expression was detected in all NSCLC tissues and the matched normal tissues. The levels of PlGF transcript in these tissues varied from weak to strong. In 17 pairs of lung cancer and normal tissue samples, PlGF mRNA expression level in cancers was significant higher than the normal tissue. There has been no report about the PlGF mRNA expression level in NSCLC, although limited reports have studied mRNA expression of VEGF, a family member of PlGF, in NSCLC. Dolrini *et al *reported VEGF mRNA expression in cancer and normal tissue samples from 22 patients with the method of quantitative competitive real time PCR [21]. They detected VEGF mRNA in 18 samples of healthy tissue and all samples of tumour tissue and found that VEGF expression was higher in the tumour tissue than in the matched healthy tissue in 17 cases [21]. The current study has also supported by a recent report to show that lung cancer cell lines expressed PlGF, although it was indicated that SCLC tissues had a higher levels of PlGF staining than NSCLC [22]. Collectively, we suggest that PlGF is a factor that has strong prognostic value in NSCLC as shown in the present study and possibly in SCLC as shown in a smaller scale study [22]. A larger scale study will undoubtedly further clarify the connection.

The other interesting finding from the current study is that PlGF mRNA expression level has no significant difference with sex, histological type, tumour size, and lymph node status. However, advanced NSCLC tumours (stage III-IV) have higher levels of PlGF expression than the early stage NSCLC (I-II). This is somewhat in contrast with the immunohistochemical assessment, in that little statistical difference was observed between different stages (table 1). This is interesting and perhaps reflects the following: (1) the sensitivity of different assays. Quantitative analysis of gene transcript is a highly sensitive technique which can detect minute amount of genetic material. The technique also allows detection of very high levels of expression. Immunohistochemical analysis and the assessment based on colorimetric staining are less sensitive and sometime subjective in nature. (2) The correlation between protein and mRNA. Although a good relationship between mRNA and protein translation in the cells have been well documented, this may not be exactly translated at the tissue level. Thus, each technique has its advantage, i.e. Q-PCR for sensitivity and quantitative nature and IHC for its ability to identify protein and the location of proteins.

In summary, the current study has shown that the angiogenic factor, PlGF is ubiquitously in lung tissues and is predominantly a cytoplasmic protein in lung epithelial and lung cancer cells. PlGF expression is significantly higher in cancer tissue than in normal tissue, and is positively correlated with tumour stage and tumour size. This indicates that PlGF may have some role in tumour progression and that blocking/targeting PlGF expression may have promising therapeutic future in NSCLC.

## Competing interests

The author(s) declare that they have no competing interests.

## Authors' contributions

**LJZ**: sample collection and preparation, study design, and patients follow-up;

**JFC**: sample processing and conducting experimental analysis;

**YK**: Experimental design and preparation of the manuscript;

**REM**: study design and manuscript preparation;

**WGJ**: study design, data analysis, and manuscript preparation.
